# Review of the Reported Measures of Clinical Validity and Clinical Utility as Arguments for the Implementation of Pharmacogenetic Testing: A Case Study of Statin-Induced Muscle Toxicity

**DOI:** 10.3389/fphar.2017.00555

**Published:** 2017-08-23

**Authors:** Marleen E. Jansen, T. Rigter, W. Rodenburg, T. M. C. Fleur, E. J. F. Houwink, M. Weda, Martina C. Cornel

**Affiliations:** ^1^Section Community Genetics, Department of Clinical Genetics and Amsterdam Public Health Research Institute, VU University Medical Center Amsterdam, Netherlands; ^2^Centre for Health Protection, National Institute for Public Health and the Environment Bilthoven, Netherlands; ^3^Division of Pharmacoepidemiology and Clinical Pharmacology, Utrecht Institute of Pharmaceutical Sciences, Utrecht University Utrecht, Netherlands

**Keywords:** clinical validity, clinical utility, pharmacogenetics, statins, translation

## Abstract

Advances from pharmacogenetics (PGx) have not been implemented into health care to the expected extent. One gap that will be addressed in this study is a lack of reporting on clinical validity and clinical utility of PGx-tests. A systematic review of current reporting in scientific literature was conducted on publications addressing PGx in the context of statins and muscle toxicity. Eighty-nine publications were included and information was selected on reported measures of effect, arguments, and accompanying conclusions. Most authors report associations to quantify the relationship between a genetic variation an outcome, such as adverse drug responses. Conclusions on the implementation of a PGx-test are generally based on these associations, without explicit mention of other measures relevant to evaluate the test's clinical validity and clinical utility. To gain insight in the clinical impact and select useful tests, additional outcomes are needed to estimate the clinical validity and utility, such as cost-effectiveness.

## Introduction

Health care professionals, scientists, and policy makers have recognized the potential of precision medicine to optimize therapeutic outcomes. Appropriate therapeutic choices could be made by prospectively identifying patients through pharmacogenetic (PGx) tests. A PGx test can identify patients at high risk of treatment failure, for example due to drug toxicity or inferior treatment efficacy. However, predictive biomarkers are not used to the expected extent in health care practice (Teng, 2011; Ratain and Johnson, 2014). Numerous barriers for the successful implementation of PGx have been described (Horgan et al., 2014; Tan-Koi et al., 2015): from lack of evidence for clinical practice to unawareness amongst health care professionals about PGx. Nonetheless, PGx is successfully applied in some health care settings, predominantly in specialized cancer care (Horgan et al., 2014). It is not always clear how decisions whether or not to implement PGx testing in a health care practice are made, and which evidence is needed for these decisions.

Several initiatives exist to translate knowledge on PGx to the broader clinical practice (Crews et al., 2012; Kapur et al., 2012). An international initiative is the Clinical Pharmacogenetics Implementation Consortium (CPIC). Currently their dosing advises based on PGx exist for over 80 drugs (Caudle et al., 2014; Relling, 2015). About one-third of these therapies are prescribed mainly by primary care providers. However, to our knowledge, no guidelines exist on how and when to test PGx markers for primary care providers. This information is also not available in the CPIC advisory documents, since it is assumed in CPIC guidelines that “clinical high-throughput and pre-emptive genotyping will eventually become common practice and clinicians will increasingly have patients'genotypes available before a presciption is written” (Caudle et al., 2014). To facilitate guideline development, policy makers need information about a range of aspects to assess the eligibility of a test for clinical practice (Dotson et al., 2016; Razum and Jahn, 2016). Two essential aspects to design guidelines are the clinical validity and utility, information that often lacks for PGx tests (Gillis and Innocenti, 2014; Janssens and Deverka, 2014). Clinical validity refers to the performance of the test, such as the discriminative ability and predictive value (Burke, 2015). Clinical utility takes assessment of the test a step further and focuses on the impact on health care, through analyses such as cost-effectiveness (Sanderson et al., 2005; Khoury et al., 2009). While for example CPIC offers a first step to bridge between PGx information from research to clinical practice, information is still needed on performance of the test and impact on care before other stakeholders will accept PGx. Medical doctors, health insurers, policy makers, but also patients need and want to know the utility of the test.

Reporting of the relevant data representing clinical validity and utility in scientific literature is currently scarce, while it is required to translate knowledge to practice. In publications, PGx is often studied through solely analyzing associations between genetic variants and either blood concentrations of the drug or health outcomes of the treatment (Button et al., 2013; Tonk et al., 2016). To provide information on the level of clinical validity of PGx, current PGx research outcomes on genotype-phenotype associations could be reassessed to calculate values to illustrate the clinical validity, such as the positive and negative predictive values (Tonk et al., 2016). Information on clinical validity would be a starting point to provide decision makers and clinicians with insight in PGx tests (Janssens and Deverka, 2014). The decision to adopt a genetic approach as part of routine care, however, is generally motivated by more than the discriminative ability of a test (Tan-Koi et al., 2015). Characteristics of clinical utility play an important role for the translation into clinical care. For example, cost-effectiveness studies, data on pilot implementation studies, and the number needed to genotype (NNTG) could enable policy makers to identify promising PGx applications (Cook and Sackett, 1995; Teng, 2011; Chan et al., 2012).

The current gap between available evidence and the lack of application of PGx needs to be explored to pursuit necessary evidence from a health care provider, patient, and policy perspective. Therefore, we aim to review scientific literature on arguments for the eligibility of PGx testing and what evidence is used to substantiate these arguments. By taking PGx for statins as an example, we focus on an often prescribed drug in primary care that has been discussed as a promising candidate for PGx. With information from recent publications on PGx for statins, we hope to facilitate the development of recommendations whether PGx is eligible for primary care. These recommendations will focus on studies needed for translation and implementation, both for statins and broader health care applications.

## Materials and methods

### Literature search and selection

A literature search in Medline was performed for publications published between 2002 and 2016. The keywords were terms related to statins, pharmacogenetics, the types of outcomes, and/or conclusions (Supplementary Box 1). All identified publications were imported into EndNote X7. Three reviewers (IH, MJ, and TR) independently screened the obtained titles and abstracts for selection based on the inclusion criteria. Only human studies published in English were included. Furthermore, publications had to address the topic of pharmacogenetics for statins and should address cardiovascular disease.

### Data extraction

In total seven reviewers (MC, TF, IH, MJ, TR, WR, MW) independently extracted data following a predefined template of relevant topics. The topics included in this template are summarized in Table 1. The data extraction was executed in phases, and results were repeatedly discussed among the research team. At least two researchers read, analyzed, and crosschecked each study. Furthermore, illustrative quotes were extracted from literature to use as examples in the results.

**Table 1:** Template of topics used to extract data from the reviewed publications.

**Topic**	**Definition**
Publication type	The type of publication, ranging from randomized controlled trial to published guidelines.
Independent variable	The statin(s) reported on, for example Simvastatin, and the genotype, such as *SLCO1B1*.
Dependent variable	The resultant outcome, such as levels of drug-efficacy.
Measures of effect	The reported outcomes, for example OR, AUC, and sensitivity.
Arguments used for or against eligibility	The reported interpretation of the authors based on their results.
Implementation advice	The suggestions of the authors to follow-up on the presented results.

*OR, odds ratio; AUC, area under the curve*.

### Analysis

From the extracted data two researchers (MJ and TR) independently analyzed the following items: (1) measures of effect; (2) arguments used for or against eligibility; and (3) implementation advice. In the data on measures of effect and arguments for eligibility we specifically analyzed: parameters for potential clinical validity, clinical validity, and clinical utility (Box 1). These constructs are dependent on each other. Without an association and sufficient prevalence of gene-variant and effect, the clinical validity will not allow for a relevant intervention. In our analysis, analytical validity was assumed, since the tests were used to evaluate clinical outcomes. To assess implementation advice (item 3), it was first evaluated if an implementation advice was given (yes/no). If an implementation advice was available, we assessed it as “positive” or “negative”; but where advice in the publications was that more research is needed, we scored it as “inconclusive.”

BOX 1 **Interpretation-guide for defining whether outcomes are expressed in terms of clinical validity or utility. These questions are derived from the ACCE-model (Sanderson et al., 2005); from Burke and Zimmern (2007) and Burke (2015). The questions include specific terms for the case of statins, where the outcome is an adverse drug response: muscle toxicity. When each item yields positive results, there is an increase in evidence for successful implementation.**

**Table:** 

**(A) Parameters for potential clinical validity**	**(B) Clinical validity**	**(C) Clinical utility**
(1) Association: What is the association between the genotype and an adverse drug response? (2) Prevalence: What is the prevalence of the relevant genotype and adverse drug response?	(1) Sensitivity: How often is the test positive when an adverse drug response is present? (2) Specificity: How often is the test negative when an adverse drug response is not present? (3) Positive predictive value: Among people with a positive test for the relevant genotype, what proportion has an adverse drug response? (4) Negative predictive value: Among people with a negative test for the relevant genotype, what proportion has an adverse drug response?	(1) Impact: What is the impact of the test result on patient care, i.e., how many adverse drug responses are caused by the genotype (PAF)? (2) Population: How many tests need to be administered for one successful intervention (NNTG)? (3) Intervention: Is there an effective remedy, acceptable action, or other measurable benefit? (4) Pilot trials: What are the results of pilot trials? (5) Health risks: What health risks can be identified for follow-up testing and/or intervention? (6) Economic: What are the economic benefits associated with actions resulting from testing?

In the next analysis step, the consistency within publications was analyzed between the three items. For example, when a publication reported *parameters for potential clinical validity* as measures of effect (item 1) and *arguments about eligibility* (item 2) it was evaluated if the implementation advice was *inconclusive*.

## Results

In total 132 publications were identified in the search, of which 89 were included for review (Supplementary Table 1). Reasons for exclusion were mainly language, a focus on drug-drug interactions instead of gene-drug interactions, description of PGx for other drugs than statins, or reporting of drug responses for other disease groups than cardiovascular disease (Supplementary Table 2).

### Descriptives

The reviewed publications included 45 original studies (RCT, cohort or case-control studies), 36 reviews, and eight expert opinions (conference report, letter to the editor, commentary or editorial). A range of statins was studied in the publications (Supplementary Table 1). Fifteen publications did not specify the statin(s) studied, but aggregated them. Simvastatin was reported in 52 publications, atorvastatin in 42. Most publications reported positive outcomes for their aim (72%), such as a statistical significant association between a genetic variant and an increase in ADRs. In the original publications (*n* = 45), the ratio of positive outcomes reported compared to the total number of studies was the largest with 35 studies that reported a positive outcome. In the review publications, a ratio of 21 to 36 was found.

### Measures of effect

In total 84 publications reported associations, prevalence, sensitivity, specificity, positive, or negative predictive value. In most publications, genotype-phenotype associations were discussed. Four publications reported on prevalence, one of these also included PPV, while another publication only reported PPV. Sensitivity and specificity were least reported; only three publications discussed both sensitivity and specificity (Supplementary Table 1), for example (Ferrari et al., 2014):

“[…] an arbitrary score based on genotype combination discriminated patients with and without CK elevation at a specificity of 97% and a sensitivity of 39%.”

Six publications discussed effects from a perspective of clinical utility such as the NNTG (Sorich et al., 2013):

“[…] approximately four individuals would need to be screened to identify one individual at higher risk of myopathy.”

Additionally, one study did not report on any of the selected measures of effect, because it was an *in vitro* study to understand influence of *SLCO1B1* polymorphism on protein function (Supplementary Table 1).

### Arguments explaining eligibility

Most authors reported on arguments for or against eligibility (Supplementary Table 1). Sixty-two publications used arguments based on associations, prevalence, sensitivity, specificity, positive or negative predictive value to explain eligibility of PGx for statins. Fifteen publications used arguments based solely on clinical utility. The arguments addressed eligibility supported by available evidence either positively (Brunham et al., 2012):

“Our findings provide further support for a role for *SLCO1B1* genotype in simvastatin-associated myopathy, and suggest that this association may be stronger for simvastatin compared with atorvastatin.”

or negatively (Leusink et al., 2016):

“Genetic variation can without a doubt affect statin response; using such information in clinical decision-making nevertheless seems far away due to the small effect sizes. Association claims regarding SNPs affecting only clinical benefit have been made although these claims rely on questionable evidence.”

Five publications applied arguments from both a clinical validity as well as clinical utility perspective. Again, these arguments could support or dispute eligibility of PGx tests for statins. In five publications the arguments for or against eligibility were not explicitly stated (Supplementary Table 1). The research presented in these publications was in early phases and focused on pharmacokinetics or the conclusions included a different intervention instead of PGx when ADRs arise, such as substituting the statin without performing a PGx test.

### Implementation advice

A limited number of authors gave a straightforward implementation advice for PGx in statin treatment (*n* = 19, Supplementary Table 1), leaning toward either positive such as (Sirtori et al., 2012):

“It seems that a systematic genetic screening for some known SNPs would most likely improve the muscle safety of the statin treatment […]”

or negative, for example (Rossi and McLeod, 2009):

“For now, the risk of severe reactions is fortunately rare and not likely to be improved by routine genetic testing.”

In the original research, eight studies gave a conclusive implementation advice. All these studies were published in the past five years (2012–2016, Supplementary Table 1), in contrast with mostly “inconclusive” advice for 2002–2011. Fourteen publications did not report strong conclusions about the implementation of PGx for statins (Supplementary Table 1), most publications advised to conduct more research (*n* = 56, Supplementary Table 1). The latter usually included discussing the potential of PGx for statins, but stressed that more research is needed before the true potential for routine care would be clear. The research mentioned could entail replication of the reported study or research aimed at implementation. However, advice lacked on when a patient should be tested also in publications that included a positive implementation advice: before or at prescription or after ADRs arise, and who should be responsible for ordering a PGx test, for example the general practitioner or the pharmacist. Specific steps to gather evidence to step through a process from scientific discovery to implementation in practice also lacked in most publications.

### Consistent reporting

Within the publications, most authors reported both measures of effect and arguments for eligibility on the level of association and prevalence (*n* = 49, Supplementary Table 1). When combining these outcomes with the implementation advice, in 15 of the publications, an implementation advice was given other than *more research needed*, i.e., inconsistent with the level of the measures of effect and arguments. In six publications no implementation advice or clear suggestions for further research were reported. In ten publications, the authors suggested alternative dosing or statin based on their publications. These implementation suggestions were not based on data on the level of clinical validity. No development towards reporting on levels of clinical validity or utility was observed in recently published publications. In fifteen publications it was specifically recognized that as the reported measure of effect were on the level of an association, arguments on clinical utility were lacking (Supplementary Table 1), for example (Ong et al., 2012):

“The implementation of pharmacogenomics for cardiovascular therapeutics on a population scale faces substantial challenges. The greatest obstacle to clinical implementation of cardiovascular pharmacogenomics may be the lack of both reproducibility and agreement about the validity and utility of the findings.”

In four publications, authors consistently reported outcome measures and arguments for eligibility both on clinical utility level, but one of these papers—from the “News section” in Nature Medicine—did not advice on implementation. In four other publications, arguments for eligibility were also reported on the level of clinical utility, but the measures of effect were on the level of (parameters of potential) clinical validity. However, none of these papers gave a positive implementation advice: three gave inconclusive advice, and one did not give an implementation advice.

Some authors commented on the research and translation process in a more general manner. These comments suggest general steps for research and translation, which apply to ensure evidence-based guidelines for PGx for statins, such as (Needham and Mastaglia, 2014):

“It is essential to first establish a valid association between a genetic variant and a drug response, which has been done for some genetic variations and muscle toxicity as reviewed above, and to then demonstrate a clinically significant outcome that results in improved patient management.”

## Discussion

While many scientific publications describe promising PGx results, implementation appears to lag behind. Our results suggest an explanation can be found in the type of evidence reported in literature, because solely associations are often reported. Other types of evidence are needed for implementation, including guideline development and coverage decisions. To review arguments for the eligibility of PGx testing and what evidence is used to substantiate this, 89 scientific publications were evaluated on PGx for statins in cardiovascular disease. In 64 of the 89 publications, positive findings were reported on the research aims. Only in nine of these 64 publications a positive implementation advice was given. As multiple authors also discuss (Ong et al., 2012; Santos et al., 2012; Ferrari et al., 2014; Kadam et al., 2016) this illustrates a lack of adequate measures of effect to conclude on implementation for clinical practice: arguments for or against the eligibility of PGx for statins cannot sufficiently be substantiated. Often the arguments put forward, are genotype-phenotype associations, and do not focus on broader implications for clinical practice. Arguments at the level of clinical validity and utility are rare, while measures such as the NNTG and PAF or cost per quality adjusted life year (QALY) are relevant for health care decision makers.

Though our study is limited to published research and is inherently influenced by publication bias, it is a unique approach to reviewing scientific publications. The broad review of arguments in publications on the topic of PGx for statins, enabled a clear identification of the gap between scientific efforts and evidence needs for implementation. The call for reporting on a level of clinical utility finds resonance in other publications that discuss the gap between reported measures and necessary measures for implementation (Voora and Ginsburg, 2012; Kapoor et al., 2016). The lack of reporting on clinical utility of PGx for statins hinders the translation of relevant tests into health care (Burke, 2015; Tonk et al., 2016). Furthermore, we did not find clear guidance in most publications to progress from solely associations to reporting additional evidence on clinical validity moving toward clinical utility. To be able to evaluate the potential of PGx for clinical practice, the evidentiary gap between publications that report associations and publications that report on clinical utility needs to be addressed.

The context in which a test should take place and be piloted remains unclear in research suggestions from current publications. The timing of a PGx test influences the impact of the test greatly, for example there are significant differences between pre-emptively testing for a broad panel or targeted testing at first prescription. One of the few international initiatives on translation of PGx information into practice—CPIC—only offers information on dosing advice (Ramsey et al., 2014). Suggestions lack on how and when to test an individual, and the assumption that the genotype is known is generally not routine clinical practice in primary care. Moreover, most of the arguments in the CPIC-guidelines are also based on evidence of associations; necessary information for decision makers on clinical utility level is for example not specified in the guideline for statins.

A bridge is needed between the necessary and highly relevant work from basic science to relevant implementation research for clinical practice. Only through following the steps from clinical validity to clinical utility in a well-defined health care context (Box 1), scientists will be able to deliver valuable PGx tests through health care providers and policy makers to the right patients. The bridge between basic science and implementation could take the shape of a common framework that attunes between the needs of stakeholders. Examples of such frameworks are available, but as this and other reviews show, incentives to follow these frameworks in translational studies seem absent (Roberts et al., 2017). Translation from bench to bedside demands implementation studies to generate evidence on the question whether specific application are eligible or not in specific settings.

## Conclusions and recommendations

Results from PGx studies on statins for cardiovascular disease are often summarized as associations between the genotype and phenotype: from effective drug levels to ADRs. For the next steps in translation, evidence is needed on clinical validity and clinical utility through implementation studies. It needs to be clearly defined what drug is studied, what the target population is, and when they should be tested. To support this contextual thinking, experts from health technology assessment could be included in early phases of research. This will offer opportunities to make recommendations on the steps from clinical validity to clinical utility, and for example include recommendation to study cost per QALY. Furthermore, to have an incentive for fundamental researchers to think about these steps, journals and peer reviewers can easily ask for measures that can directly be derived from measures for association. As Tonk et al. (2016) described, measures for disease frequency and prevalence of exposure can be calculated from the data needed for associations, and as such PAF, PPV, NPV, and NNTG can be reported on. To bring these recommendations toward clinical utility into practice, pilot studies should be financed. Translation for interventions should be focused on as a return on investment in subsidies from financers. Roles and responsibilities in the research field need to bridge between efforts in research and needs in practice to bring PGx with proven clinical utility to the patient.

## Author contributions

MJ contributed to the analysis and interpretation of data, drafting the work and revising it critically. TR, WR, and MC contributed to the conception of the work, analysis and interpretation of data, and revising the work critically. TF, MW contributed to the analysis and interpretation of data, and revising the work critically. EH contributed to the conception of the work, and the data analysis. All authors approved the version to be published.

### Conflict of interest statement

The authors declare that the research was conducted in the absence of any commercial or financial relationships that could be construed as a potential conflict of interest.

## References

[B1] Brunham L. R. Lansberg P. J. Zhang L. Miao F. Carter C. Hovingh G. K.. (2012). Differential effect of the rs4149056 variant in SLCO1B1 on myopathy associated with simvastatin and atorvastatin. Pharmacogenomics J. 12, 233–237. 10.1038/tpj.2010.92 21243006

[B2] Burke W. (2015). Genetic tests: clinical validity and clinical utility. Curr. Protoc. Human Genet. 81, 9.15. 11–19.15. 18. 10.1002/0471142905.hg0915s81 24763995 PMC4084965

[B3] Burke W. Zimmern R. (2007). Moving Beyond Acce: An Expanded Framework for Genetic Test Evaluation. A Paper for the United kingdom Genetic Testing Network. Available online at: http://www.phgfoundation.org/file/16270 (Accessed April 25, 2017).

[B4] Button K. S. Ioannidis J. P. Mokrysz C. Nosek B. A. Flint J. Robinson E. S.. (2013). Power failure: why small sample size undermines the reliability of neuroscience. Nat. Rev. Neurosci. 14, 365–376. 10.1038/nrn3475 23571845

[B5] Caudle K. E. Hoffman J. M. Whirl-Carrillo M. Haidar C. E. Crews K. R. Klein T. E. (2014). The clinical pharmacogenetics implementation consortium (CPIC): Facilitating the adoption of pharmacogenetics into routine clinical practice and the electronic health record. Pharmacotherapy 34, e251–e252.

[B6] Chan S. L. Suo C. Chia K. S. Teo Y. Y. (2012). The population attributable fraction as a measure of the impact of warfarin pharmacogenetic testing. Pharmacogenomics 13, 1247–1256. 10.2217/pgs.12.104 22920395

[B7] Cook R. J. Sackett D. L. (1995). The number needed to treat: a clinically useful measure of treatment effect. BMJ 310:452. 10.1136/bmj.310.6977.452 7873954 PMC2548824

[B8] Crews K. R. Hicks J. K. Pui C. H. Relling M. V. Evans W. E. (2012). Pharmacogenomics and individualized medicine: translating science into practice. Clin. Pharmacol. Ther. 92, 467–475. 10.1038/clpt.2012.120 22948889 PMC3589526

[B9] Dotson W. D. Bowen M. S. Kolor K. Khoury M. J. (2016). Clinical utility of genetic and genomic services: context matters. Genet. Med. 18, 672–674. 10.1038/gim.2015.153 26656648 PMC4902786

[B10] Ferrari M. Guasti L. Maresca A. Mirabile M. Contini S. Grandi A. M.. (2014). Association between statin-induced creatine kinase elevation and genetic polymorphisms in SLCO1B1, ABCB1 and ABCG2. Eur. J. Clin. Pharmacol. 70, 539–547. 10.1007/s00228-014-1661-6 24595600

[B11] Gillis N. K. Innocenti F. (2014). Evidence required to demonstrate clinical utility of pharmacogenetic testing: the debate continues. Clin. Pharmacol. Ther. 96, 655–657. 10.1038/clpt.2014.185 25399714

[B12] Horgan D. Jansen M. Leyens L. Lal J. A. Sudbrak R. Hackenitz E.. (2014). An index of barriers for the implementation of personalised medicine and pharmacogenomics in Europe. Public Health Genomics 17, 287–298. 10.1159/000368034 25401385

[B13] Janssens A. C. Deverka P. A. (2014). Useless until proven effective: the clinical utility of preemptive pharmacogenetic testing. Clin. Pharmacol. Ther. 96, 652–654. 10.1038/clpt.2014.186 25399713

[B14] Kadam P. Ashavaid T. F. Ponde C. K. Rajani R. M. (2016). Genetic determinants of lipid-lowering response to atorvastatin therapy in an Indian population. J. Clin. Pharm. Ther. 41, 329–333. 10.1111/jcpt.12369 26932749

[B15] Kapoor R. Tan-Koi W. C. Teo Y.-Y. (2016). Role of pharmacogenetics in public health and clinical health care: a SWOT analysis. Eur. J. Hum. Genet. 24, 1651–1657. 10.1038/ejhg.2016.114 27577547 PMC5117918

[B16] Kapur S. Phillips A. G. Insel T. R. (2012). Why has it taken so long for biological psychiatry to develop clinical tests and what to do about it? Mol. Psychiatr. 17, 1174–1179. 10.1038/mp.2012.105 22869033

[B17] Khoury M. J. McBride C. M. Schully S. D. Ioannidis J. P. Feero W. G. Janssens A. C.. (2009). The Scientific Foundation for personal genomics: recommendations from a national institutes of health-centers for disease control and prevention multidisciplinary workshop. Genet. Med. 11, 559–567. 10.1097/GIM.0b013e3181b13a6c 19617843 PMC2936269

[B18] Leusink M. Onland-Moret N. C. de Bakker P. I. de Boer A. Maitland-van der Zee A. H. (2016). Seventeen years of statin pharmacogenetics: a systematic review. Pharmacogenomics 17, 163–180. 10.2217/pgs.15.158 26670324

[B19] Needham M. Mastaglia F. L. (2014). Statin myotoxicity: a review of genetic susceptibility factors. Neuromuscul. Disord. 24, 4–15. 10.1016/j.nmd.2013.09.011 24176465

[B20] Ong F. S. Deignan J. L. Kuo J. Z. Bernstein K. E. Rotter J. I. Grody W. W.. (2012). Clinical utility of pharmacogenetic biomarkers in cardiovascular therapeutics: a challenge for clinical implementation. Pharmacogenomics 13, 465–475. 10.2217/pgs.12.2 22380001 PMC3306231

[B21] Ramsey L. B. Johnson S. G. Caudle K. E. Haidar C. E. Voora D. Wilke R. A.. (2014). The clinical pharmacogenetics implementation consortium guideline for SLCO1B1 and simvastatin-induced myopathy: 2014 update. Clin. Pharmacol. Ther. 96, 423–428. 10.1038/clpt.2014.125 24918167 PMC4169720

[B22] Ratain M. J. Johnson J. A. (2014). Meaningful use of pharmacogenetics. Clin. Pharmacol. Ther. 96, 650–652. 10.1038/clpt.2014.188 25399712

[B23] Razum O. Jahn A. (2016). Molecular and genomic sciences in health: apply the established rules of evidence. Int. J. Public Health 61, 405–407. 10.1007/s00038-015-0755-y 26496902

[B24] Relling M. (2015). Clinical implementation of pharmacogenetics: CPIC guidelines. Clin. Chem. Lab. Med. 53:S75.

[B25] Roberts M. C. Kennedy A. E. Chambers D. A. Khoury M. J. (2017). The current state of implementation science in genomic medicine: opportunities for improvement. Genet. Med. 19, 858–863. 10.1038/gim.2016.210 28079898 PMC6312663

[B26] Rossi J. S. McLeod H. L. (2009). The pharmacogenetics of statin therapy: when the body aches, the mind will follow. J. Am. Coll. Cardiol. 54, 1617–1618. 10.1016/j.jacc.2009.06.027 19833261

[B27] Sanderson S. Zimmern R. Kroese M. Higgins J. Patch C. Emery J. (2005). How can the evaluation of genetic tests be enhanced? Lessons learned from the ACCE framework and evaluating genetic tests in the United Kingdom. Genet. Med. 7, 495–500. 10.1097/01.gim.0000179941.44494.73 16170241

[B28] Santos P. C. Gagliardi A. C. Miname M. H. Chacra A. P. Santos R. D. Krieger J. E. (2012). SLCO1B1 haplotypes are not associated with atorvastatin-induced myalgia in Brazilian patients with familial hypercholesterolemia. Eur. J. Clin. Pharmacol. 68, 273–279. 10.1007/s00228-011-1125-1 21928084

[B29] Sirtori C. R. Mombelli G. Triolo M. Laaksonen R. (2012). Clinical response to statins: mechanism(s) of variable activity and adverse effects. Ann. Med. 44, 419–432. 10.3109/07853890.2011.582135 21623698

[B30] Sorich M. J. Wiese M. D. O'Shea R. L. Pekarsky B. (2013). Review of the cost effectiveness of pharmacogenetic-guided treatment of hypercholesterolaemia. Pharmacoeconomics 31, 377–391. 10.1007/s40273-013-0045-6 23568333

[B31] Tan-Koi W. C. Kapoor R. Teo Y. Y. (2015). Pharmacogenetics through a public health lens: from policy to practice. Pharmacogenet. Genomics 25, 518–520. 10.1097/FPC.0000000000000159 26325520

[B32] Teng K. (2011). Pharmacogenomics for the primary care provider: why should we care? Cleve. Clin. J. Med. 78, 241–242. 10.3949/ccjm.78a.11017 21460129

[B33] Tonk E. C. Gurwitz D. Maitland-van der Zee A. H. Janssens A. C. (2016). Assessment of pharmacogenetic tests: presenting measures of clinical validity and potential population impact in association studies. Pharmacogenomics J. 17, 386–392. 10.1038/tpj.2016.34 27168098 PMC5549182

[B34] Voora D. Ginsburg G. S. (2012). Clinical application of cardiovascular pharmacogenetics. J. Am. Coll. Cardiol. 60, 9–20. 10.1016/j.jacc.2012.01.067 22742397

