# Supplementary material for: Review of the Reported Measures of Clinical Validity and Clinical Utility as Arguments for the Implementation of Pharmacogenetic Testing: A Case Study of Statin-Induced Muscle Toxicity
Source: Front Pharmacol. 2017 Aug 23;8:555. doi: 10.3389/fphar.2017.00555 (PMC5572384; doi:10.3389/fphar.2017.00555)
Supplement: Supplementary file 1 [file Table1.docx]

**SUPPLEMENTARY MATERIAL**

**Arguments for eligibility of pharmacogenetic testing in scientific literature: statins as a case study**

Jansen ME^1,2^, Rigter T^1,2^, Rodenburg W^2^, Fleur TMC^1,3^, Houwink EJF^1^, Weda M^2^, Cornel MC^1^

1 Department of Clinical Genetics, section Community Genetics and Amsterdam Public Health research institute, VU University Medical Center, Amsterdam, The Netherlands

2 Centre for Health Protection, National Institute for Public Health and the Environment, Bilthoven, The Netherlands.

3 Utrecht Institute of Pharmaceutical Sciences, Division of Pharmacoepidemiology & Clinical Pharmacology, Utrecht University, Utrecht, The Netherlands

M.E. Jansen, PhD, Amsterdam Public Health research institute, Community Genetics and Public Health Genomics, P.O. Box 7057, BS7/A509, 1007 MB Amsterdam, The Netherlands.

T +31 (0)20 444 8914 | F +31 (0)20 444 8665

[m.jansen5@vumc.nl](mailto:m.jansen5@vumc.nl)

**Supplementary box 1.** Search strategy for MEDLINE database, searched between 2002 to 2016.

Database: MEDLINE 2002 to 2016, MEDLINE In-Process & Other Non-Indexed Citations

Search Strategy:

--------------------------------------------------------------------------------

1 (SLCO1B1* or SORT1 or CELSR2 or PSRC1).tw. or (SLCO1B1* or SORT1 or CELSR2 or PSRC1).kw. (557)

2 (SLCO1b1 protein, human or CELSR2 protein, human or PSRC1 protein, human).rn. (479)

3 1 or 2 (800)

4 (simvastatin or pravastatin or atorvastatin or fluvastatin).tw. or (simvastatin or pravastatin or atorvastatin or fluvastatin).kw. (15143)

5 simvastatin/ or pravastatin/ or (atorvastatin or fluvastatin).rn. (13704)

6 4 or 5 (17667)

7 3 and 6 (160)

8 statins.ti. or statins.kw. or (cardiovascular agent*.ti. or *cardiovascular agents/) (12599)

9 hydroxymethylglutaryl-CoA reductase inhibitors/ (22622)

10 8 or 9 (30072)

11 3 and 10 (156)

12 *pharmacogenetics/ or (pharmacogenetic* or personali?ed or individual or interindividual).ti. (48660)

13 3 and 12 (70)

14 7 or 11 or 13 (264)

15 ("quality of life" or morbidity or mortality or cost effectiveness or treatment costs or treatment outcome or patient-relevant outcome or treatment efficay or treatment effectiveness or treatment benefit* or clinical utility or clincial efficacy or clinical effectiveness or clincal benefit* or Sensitivity, Specificity, PPV, NPV or evidence or rationale or "sufficiently robust" or tolerance or tolerability).tw. (2226443)

16 (effectiv* or ineffect*).tw. or (efficacy or benefit*or utility or cost*).ti. (1509115)

17 "quality of life"/ or morbidity/ or mortality/ or risk assessment/ or risk factors/ or drug monitoring/ or cost-benefit analysis/ or health care costs/ or treatment outcome/ or drug tolerance/ (1596095)

18 (adverse effects or adverse events or "side effects").tw. or ae.fs. (1634993)

19 14 and (15 or 16 or 17 or 18) (132)

20 exp animals/ not (exp animals/ and humans/) (4078149)

21 ("in vitro" or "ex vivo").ti. or ("in vitro" or "ex vivo").kw. (258520)

22 (rodent* or rat* or mouse or mice or murine or ferret* or pig* or guineapi* or hamster* or rabbit* or mammalian or monkey* or dog or cacine or zebrafish or animal model*).ti. (1878003)

23 19 not (20 or 21 or 22) (128)

24 limit 23 to yr=1995-2015 (128)

25 remove duplicates from 24 (119)

Supplementary Table 1. Analysis of measures of effect, arguments and implementation advice reported in the reviewed articles. Definitions of terms are available in Box 1.

| **Author** | **Statins studied** | **Type study** | **Measures of effect** | **Positive outcome** | **Arguments for or against eligibility** | **Implementation advice** |
| --- | --- | --- | --- | --- | --- | --- |
| ***Original research*** |  |  |  |  |  |  |
| Hedman, 2003^1^ | Pravastatin | Cohort study | Association | Yes | Association | Inconclusive |
| Hermann, 2006^2^ | Atorvastatin | Case control | Association | No | Association | None |
| Igel, 2006^3^ | Pravastatin | Case control | Association | Yes | Association | Inconclusive |
| Pasanen, 2006^4^ | Simvastatin | Cohort study | Association | Yes | Association; Prevalence | Inconclusive |
| Zhang, 2007^5^ | Pravastatin | Cohort study | Association | Yes | None | Inconclusive |
| Couvert, 2008^6^ | Fluvastatin | RCT | Association | Yes | Intervention | Inconclusive |
| Link, 2008^7^ | Simvastatin | Case control | Association; Prevalence | Yes | Intervention | Inconclusive |
| Furihata, 2009^8^ | Pravastatin | In vitro study | Other | Yes | Association | None |
| Voora, 2009^9^ | Atorvastatin, Simvastatin, Pravastatin | RCT | Association | Yes | Association | Inconclusive |
| Peters, 2010^10^ | Any statin, mostly Simvastatin, Pravastatin and Atorvastatin | Case control | Association | No | Association | Inconclusive |
| Toms, 2010^11^ | Not specified, but at least Simvastatin and Atorvastatin | Case-control | Association | Yes | Association | Inconclusive |
| Donnelly, 2011^12^ | Simvastatin, Atorvastatin, Pravastatin, Fluvastatin, Cerivastatin, Rosuvastatin | Cohort study | Association | Yes | Association | Inconclusive |
| Marciante, 2011^13^ | Cases: All Cerivastatin,  Controls: Lovastatin, Simvastatin, Atorvastatin, Fluvastatin, Pravastatin, or Cerivastatin | Case control | Association | Yes | Association | None |
| Rodrigues, 2011^14^ | Atorvastatin | Cohort | Association | Yes | PPV, NPV | Inconclusive |
| Santos, 2011^15^ | None: frequencies of genotypes observed in populations | Cross-sectional study | Association | Yes | Impact | Inconclusive |
| Kaddurah-Daouk, 2011^16^ | Simvastatin | Nested-case-control | Association | Yes | PPV, NPV | Inconclusive |
| Trompet, 2011^17^ | Pravastatin | GWAS | Association | Yes | Association | None |
| Yang, 2011^18^ | Simvastatin | Case study | Association | Yes | Association | Inconclusive |
| Akao, 2012^19^ | Pravastatin | RCT | Association | Yes | Association | None |
| Brunham, 2012^20^ | Simvastatin, Atorvastatin, Pravastatin, Rosuvastatin | Case control study | Association | Yes | Association | Inconclusive |
| Francesca, 2012^21^ | Atorvastatin | Case study | Association | Yes | Association | Yes, positive |
| Hubacek, 2012^22^ | Simvastatin, Atorvastatin. | Case-control | Association | Yes | Association; Prevalence | Inconclusive |
| Santos, 2012^23^ | Atorvastatin | Case-control | Association | No | Association | Inconclusive |
| Sortica, 2012^24^ | Simvastatin | Cohort study | Association | Yes | Association | Inconclusive |
| Vrablik, 2012^25^ | Simvastatin, Atorvastatin, Rosuvastatin | Case control | Association | No | Association | Inconclusive |
| Carr, 2013^26^ | Simvastatin, Atorvastatin and other statins (<10% of patients and controls) | Case control | Association | Yes | Association | Yes, positive |
| Danik, 2013^27^ | Rosuvastatin | RCT | Association | No | Association | Yes, negative |
| Fu, 2013^28^ | Atorvastatin and Rosuvastatin | Cohort study | Association | No | Association | Yes, negative |
| Hopewell, 2013^29^ | Simvastatin | RCT | Association | Yes | Sensitivity, Specificity, PPV, NPV | Yes, negative |
| Shabana, 2013^30^ | Atorvastatin | Cohort study | Association | No | Association | Inconclusive |
| Tamraz, 2013^31^ | Cerivastatin | Nested cross sectional study | Association | Yes | Association | Inconclusive |
| de Keyser, 2014^32^ | Simvastatin and Atorvastatin | Cohort study | Association | Yes | Association | Yes, positive |
| Drobny, 2014^33^ | Primarily fluvastatin and rosuvastatin | Nested cross sectional study | Association | Yes | None | None |
| Ferrari, 2014^34^ | Atorvastatin, Rosuvastatin, simvastatin | Case-control | Association; Sensitivity; Specificity | Yes | Association; Sensitivity; Specificity | Inconclusive |
| Li, 2014^35^ | Simvastatin, Atorvastatin, Pravastatin, Rosuvastatin | Case-control | Intervention; Pilot trials | Yes | Intervention; Pilot trials | Yes, positive |
| Tsamandouras, 2014^36^ | Simvastatin | Cohort study | Association | Yes | Association | Inconclusive |
| Birmingham, 2015a^37^ | Rosuvastatin, Atorvastatin, Simvastatin | RCT | Association | Yes | Association | Inconclusive |
| Birmingham, 2015b^38^ | Rosuvastatin | Case control | Association | Yes | Association | Inconclusive |
| Shim, 2015^39^ | Simvastatin & Pravastatin | GWAS | Association | Yes | None | None |
| Hubacek, 2015^40^ | Simvastatin and Atorvastatin | Case-control | Association | No | Association | Inconclusive |
| Meyer, 2015^41^ | Simvastatin, Pravastatin, Lovastatin, Fluvastatin, Atorvastatin | Cohort study | Association | Yes | Association | Inconclusive |
| Nagy, 2015^42^ | None | Observational | Prevalence | Yes | Prevalence | Inconclusive |
| Prado, 2015^43^ | Atorvastatin | Observational | Association | No | Association | Inconclusive |
| Kadam, 2016^44^ | Atorvastatin | Cohort study | Association | Yes | None | Inconclusive |
| Sychev, 2016^45^ | None | Observational | Prevalence | Yes | Prevalence | Yes, positive |
| ***Reviews*** |  |  |  |  |  |  |
| Romaine, 2010^46^ | Various | Review | Association | No | Association | Inconclusive |
| Generaux, 2011^47^ | Atorvastatin, Cerivastatin, Fluvastatin, Lovastatin, Pitavastatin, Pravastatin, Rosuvastatin, Simvastatin | Review | Association | Yes | Association | Yes, positive |
| Maggo, 2011^48^ | Prodrug lactone form: Simvastatin, Lovastatin.  Active acid form: Atorvastatin, Fluvastatin, Pravastatin, Rosuvastatin, Pitavastatin | Review | Association | Yes | Lack of insight in Economic | Inconclusive |
| Wang, 2011^49^ | Atorvastatin; Lovastatin; Simvastatin; Rosuvastatin; Fluvastatin; Pravastatin | Review | Association | No | Lack of Association | Inconclusive |
| Cardelli, 2012^50^ | Pravastatin; Simvastatin; Atorvastatin | Review | Prevalence | No | Lack of insight in all factors | Inconclusive |
| Daly, 2012^51^ | Simvastatin | Review | Association | Yes | Lack of Sensitivity and Specificity | Inconclusive |
| Feng, 2012^52^ | Statins in general | Review | Association | No | Association | Inconclusive |
| Ong, 2012^53^ | Simvastatin, Atorvastatin, Cerivastatin | Review | Association | Yes | Lack of insight in PPV and NPV | Inconclusive |
| Scarpini, 2012^54^ | Simvastatin, Pravastatin, Atorvastatin, Cerivastatin, Rosuvastatin, Lovastatin | Review | Association | No | Association | Inconclusive |
| Sirtori, 2012^55^ | Atorvastatin, Fluvastatin, Rosuvastatin, Lovastatin, Simvastatin, Pravastatin, Pitavastatin | Review | Association | Yes | Association | Yes, positive |
| Voora, 2012^56^ | Simvastatin, Atorvastatin, Pravastatin, Rosuvastatin, Fluvastatin | Review | Association | No | Association | Inconclusive |
| Wells, 2012^57^ | Simvastatin, Atorvastatin, Lovastatin | Review | Association , PPV | Yes | Association, PPV | Yes, positive |
| Wilke 2012^58^ | Simvastatin | Review/CPIC guideline | Association , Prevalence , Population | Yes | Association , Prevalence , Population | Yes, positive |
| Johnson 2013^59^ | Simvastatin | Review | Association | Yes | Association and lack of insight in Impact, Population, Intervention, Pilot trials, Health risks, Economic | Inconclusive |
| Kitzmiller 2013^60^ | Atorvastatin, Simvastatin, Rosuvastatin, Pravastatin, Fluvastatin, Lovastatin, Pitavastatin, Cerivastatin. | Review | Association | Yes | Association , Impact | Yes, negative |
| Sorich 2013^61^ | Grouped ("statins") | Review | Population (C2) | No | Lack of insight in Impact, Population, Intervention, Pilot trials, Health risks, Economic | Yes |
| Stewart 2013^62^ | Simvastatin | Review | Association , Prevalence , Sensitivity, Specificity, PPV, NPV (B1-4) | No | Lack of insight in Impact, Population, Intervention, Pilot trials, Health risks, Economic | None |
| Yip 2013^63^ | Grouped ("statins") | Review | Association | Yes | Lack of insight in Impact, Population, Intervention, Pilot trials, Health risks, Economic | Inconclusive |
| Desai 2014^64^ | Simvastatin, Atorvastatin, Cerivastatin | Review | Association | Yes | Lack of insight in Impact, Population, Intervention, Pilot trials, Health risks, Economic | Inconclusive |
| Gelissen 2014^65^ | Lovastatin, Simvastatin, Pravastatin, Fluvastatin, Atorvastatin, Rosuvastatin, Pitavastatin | Review | Association | Yes | Economic (C6) | None |
| Hopewell 2014^66^ | Grouped ("statins") | Review | Association | No | Lack of insight in Impact, Population, Intervention, Pilot trials, Health risks, Economic | Inconclusive |
| Needham 2014^67^ | Grouped ("statins") | Review | Association | Yes | Lack of insight in Impact, Population, Intervention, Pilot trials, Health risks, Economic | Inconclusive |
| Norata 2014^68^ | Simvastatin, Lovastatin, Pravastatin, Atorvastatin, Fluvastatin, Rosuvastatin, Pitavastatin | Review | Association | No | Lack of Association | Inconclusive |
| Postmus 2014^69^ | Grouped ("statins": not stratified for statin type) | GWAS meta-analysis | Association | Yes | Lack of insight in Sensitivity, Specificity, PPV, NPV and Impact, Population, Intervention, Pilot trials, Health risks, Economic | Inconclusive |
| Ramsey, 2014^70^ | Simvastatin | Systematic literature review | Association | Yes | Association and lack of insight in Sensitivity, Specificity, PPV, NPV | Yes, positive |
| Sissung, 2014^71^ | Atorvastatin, Cerivastatin, Gluvastatin, Rosuvastatin, Simvastatin, Pitavastatin, Pravastatin | Review | Association | Yes | Lack of insight in PPV and NPV | Inconclusive |
| Tompkins, 2014^72^ | Simvastatin, Lovastatin, Fluvastatin, Rosuvastatin, Pitavastatin, Pravastatin, Cerivastatin, Atorvastatin | Review | Association | Yes | Unclear | None |
| Turner, 2014^73^ | Simvastatin, Pravastatin, Rosuvastatin, Atorvastatin | Review | Association | No | Lack of insight in Impact, Population, Intervention, Pilot trials, Health risks, Economic | None |
| Weeke, 2014^74^ | Mainly Pravastatin and Simvastatin | Review | Association | Yes | Association; Prevalence | Yes, positive |
| Dai 2015^75^ | Various | Review | Association | No | Association | Inconclusive |
| Dou, 2015^76^ | Pravastatin, Fluvastatin, Simvastatin, Rosuvastatin, Pitavastatin, Atorvastatin | Meta-analysis | Association | Yes | Association | Inconclusive |
| Hou, 2015^77^ | Various | Meta-analysis | Association | Yes | Association | Inconclusive |
| Patel 2015^78^ | Various | Review | Association | Yes | Association; Prevalence | Inconclusive |
| Kitzmiller 2016^79^ | Various | Review | Association | No | Association | Inconclusive |
| Leusink, 2016^80^ | Grouped into "statins" | Review | Association | Yes | Lack of insight in Association | Inconclusive |
| Tonk 2016^81^ | Simvastatin | Review | Association; Prevalence; Sensitivity; Specificity; PPV, NPV, Impact; Population | No | Lack of insight in Sensitivity; Specificity; PPV, NPV, Impact; Population | Yes, negative |
| ***Expert opinion*** |  |  |  |  |  |  |
| Nakamura, 2008^82^ | Simvastatin; Pravastatin | Commentary | PPV | Yes | PPV | Inconclusive |
| Mitka, 2009^83^ | Simvastatin | Editorial | Economic | No | Population, Health risks | Yes, negative |
| Rossi, 2009^84^ | Simvastatin; Pravastatin; Atorvastatin | Commentary | Association | No | Prevalence | Yes, negative |
| Vladutiu, 2009^85^ | Simvastatin | Letter to the editor | Association | No | Association | No |
| Puccetti, 2010^86^ | Rosuvastatin and Atorvastatin | Letter to the editor | Association | Yes | Association | Inconclusive |
| Becquemont, 2011^87^ | Simvastatin and Fluvastatin | Summary of conference | Association | Yes | Association | Inconclusive |
| Couvert, 2011^88^ | Grouped into "statins" | Editorial | Prevalence and PPV | Yes | PPV, NPV, Economic | Inconclusive |
| Dolgin, 2013^89^ | Grouped into "statins" | "News" | Impact, Population, Intervention | Yes | Impact, Population | None |

Supplementary Table 2. Excluded articles based on title and abstract.

| **Author(s)** | **Title** | **Year** | **Details for exclusion** |
| --- | --- | --- | --- |
| *Language* | | | |
| Rosskopf D; Meyer zu Schwabedissen HE; Kroemer HK; Siegmund W.^90^ | Pharmacogenomics in routine medical care | 2010 | German |
| Dendramis G.^91^ | Interindividual differences in the response to statin therapy and gene polymorphisms related to myopathy during statin therapy | 2011 | Italian |
| Saito Y; Sawada J.^92^ | Pharmacogenomics | 2009 | Japanese |
| Chiba K; Morimoto K.^93^ | Genetic marker of statin-induced rhabdomyolysis | 2011 | Japanese |
| Garcia-Sabina A; Gulin-Davila J; Sempere-Serrano P; Gonzalez-Juanatey C; Martinez-Pacheco R.^94^ | Specific considerations on the prescription and therapeutic interchange of statins | 2012 | Spanish |
| Garcia-Estevez DA; San Millan B; Navarro C; Sogo T.^95^ | Myopathy due to deficiency of desaminase myoadenilate induced by atorvastatine | 2013 | Spanish |
| Garwicz D; Wadelius M.^96^ | Pharmacogenetic analysis can predict adverse effects of statins | 2013 | Swedish |
| *Unrelated topic* | | | |
| Anonymous.^97^ | Drug interactions with simvastatin. | 2008 | Drug-drug interaction |
| Deng JW; Shon JH; Shin HJ; Park SJ; Yeo CW; Zhou HH; Song IS; Shin JG.^98^ | Effect of silymarin supplement on the pharmacokinetics of rosuvastatin. | 2008 | Drug-drug interaction |
| Shin KH; Kim TE; Kim SE; Lee MG; Song IS; Yoon SH; Cho JY; Jang IJ; Shin SG; Yu KS.^99^ | The effect of the newly developed angiotensin receptor II antagonist fimasartan on the pharmacokinetics of atorvastatin in relation to OATP1B1 in healthy male volunteers. | 2011 | Drug-drug interaction |
| Elsby R; Hilgendorf C; Fenner K.^100^ | Understanding the critical disposition pathways of statins to assess drug-drug interaction risk during drug development: it's not just about OATP1B1. | 2012 | Drug-drug interaction |
| Hua WJ; Hua WX; Fang HJ.^101^ | The role of OATP1B1 and BCRP in pharmacokinetics and DDI of novel statins | 2012 | Drug-drug interaction |
| Marusic S; Lisicic A; Horvatic I; Bacic-Vrca V; Bozina N.^102^ | Atorvastatin-related rhabdomyolysis and acute renal failure in a genetically predisposed patient with potential drug-drug interaction. | 2012 | Drug-drug interaction |
| Custodio JM; Wang H; Hao J; Lepist EI; Ray AS; Andrews J; Ling KH; Cheng A; Kearney BP; Ramanathan S.^103^ | Pharmacokinetics of cobicistat boosted-elvitegravir administered in combination with rosuvastatin. | 2014 | Drug-drug interaction |
| Kellick KA; Bottorff M; Toth PP; The National Lipid Association's Safety Task Force.^104^ | A clinician's guide to statin drug-drug interactions. | 2014 | Drug-drug interaction |
| Kulmatycki, K., Hanna, I., Meyers, D., Salunke, A., Movva, A., Majumdar, T., . . . Chen, J.^105^ | Evaluation of a potential transporter-mediated drug interaction between rosuvastatin and pradigastat, a novel DGAT-1 inhibitor. | 2015 | Drug-drug interaction |
| Li, D. Q., Kim, R., McArthur, E., Fleet, J. L., Bailey, D. G., Juurlink, D., . . . Garg, A. X.^106^ | Risk of adverse events among older adults following co-prescription of clarithromycin and statins not metabolized by cytochrome P450 3A4. | 2015 | Drug-drug interaction |
|  | | | |
| Howe K; Sanat F; Thumser AE; Coleman T; Plant N.^107^ | The statin class of HMG-CoA reductase inhibitors demonstrate differential activation of the nuclear receptors PXR, CAR and FXR, as well as their downstream target genes. | 2011 | Other determinant |
| Bjorkhem-Bergman L; Bergstrom H; Johansson M; Parini P; Eriksson M; Rane A; Ekstrom L.^108^ | Atorvastatin treatment induces uptake and efflux transporters in human liver. | 2013 | Other determinant |
| Hu M; Mak VW; Yin OQ; Chu TT; Tomlinson B.^109^ | Effects of grapefruit juice and SLCO1B1 388A>G polymorphism on the pharmacokinetics of pitavastatin. | 2013 | Other determinant |
| Stojakovic N; Igic R.^110^ | Simvastatin-lnduced nocturnal leg pain disappears with pravastatin substitution. | 2013 | Other determinant |
| Apostolopoulou M; Corsini A; Roden M.^111^ | The role of mitochondria in statin-induced myopathy. | 2015 | Other determinant |
|  | | | |
| Werner D; Werner U; Meybaum A; Schmidt B; Umbreen S; Grosch A; Lestin HG; Graf B; Zolk O; Fromm MF.^112^ | Determinants of steady-state torasemide pharmacokinetics: impact of pharmacogenetic factors, gender and angiotensin II receptor blockers. | 2008 | Other disease group |
| Han JY; Lim HS; Park YH; Lee SY; Lee JS.^113^ | Integrated pharmacogenetic prediction of irinotecan pharmacokinetics and toxicity in patients with advanced non-small cell lung cancer | 2009 | Other disease group |
| Innocenti F; Kroetz DL; Schuetz E; Dolan ME; Ramirez J; Relling M; Chen P; Das S; Rosner GL; Ratain MJ.^114^ | Comprehensive pharmacogenetic analysis of irinotecan neutropenia and pharmacokinetics. | 2009 | Other disease group |
| Ni W; Ji J; Dai Z; Papp A; Johnson AJ; Ahn S; Farley KL; Lin TS; Dalton JT; Li X; Jarjoura D; Byrd JC; Sadee W; Grever MR; Phelps MA.^115^ | Flavopiridol pharmacogenetics: clinical and functional evidence for the role of SLCO1B1/OATP1B1 in flavopiridol disposition. | 2010 | Other disease group |
| Di Paolo A; Bocci G; Polillo M; Del Re M; Di Desidero T; Lastella M; Danesi R.^116^ | Pharmacokinetic and pharmacogenetic predictive markers of irinotecan activity and toxicity | 2011 | Other disease group |
| Yimer G; Ueda N; Habtewold A; Amogne W; Suda A; Riedel KD; Burhenne J; Aderaye G; Lindquist L; Makonnen E; Aklillu E.^117^ | Pharmacogenetic & pharmacokinetic biomarker for efavirenz based ARV and rifampicin based anti-TB drug induced liver injury in TB-HIV infected patients. | 2011 | Other disease group |
| Kile DA; MaWhinney S; Aquilante CL; Rower JE; Castillo-Mancilla JR; Anderson PL.^118^ | A population pharmacokinetic-pharmacogenetic analysis of atazanavir. | 2012 | Other disease group |
| Shiotani A; Murao T; Sakakibara T; Tarumi K; Manabe N; Kamada T; Kusunoki H; Haruma K.^119^ | Association of SLCO1B1 1b with peptic ulcer amongst Japanese patients taking low-dose aspirin. | 2012 | Other disease group |
| De Mattia E; Toffoli G; Polesel J; D'Andrea M; Corona G; Zagonel V; Buonadonna A; Dreussi E; Cecchin E.^120^ | Pharmacogenetics of ABC and SLC transporters in metastatic colorectal cancer patients receiving first-line FOLFIRI treatment. | 2013 | Other disease group |
| Huang L; Zhang T; Xie C; Liao X; Yu Q; Feng J; Ma H; Dai J; Li M; Chen J; Zang A; Wang Q; Ge S; Qin K; Cai J; Yuan X.^121^ | SLCO1B1 and SLC19A1 gene variants and irinotecan-induced rapid response and survival: a prospective multicenter pharmacogenetics study of metastatic colorectal cancer. | 2013 | Other disease group |
| Radtke S; Zolk O; Renner B; Paulides M; Zimmermann M; Moricke A; Stanulla M; Schrappe M; Langer T.^122^ | Germline genetic variations in methotrexate candidate genes are associated with pharmacokinetics, toxicity, and outcome in childhood acute lymphoblastic leukemia. | 2013 | Other disease group |
| Han N; Yun HY; Kim IW; Oh YJ; Kim YS; Oh JM.^123^ | Population pharmacogenetic pharmacokinetic modeling for flip-flop phenomenon of enteric-coated mycophenolate sodium in kidney transplant recipients. | 2014 | Other disease group |
| Nishijima T; Tsuchiya K; Tanaka N; Joya A; Hamada Y; Mizushima D; Aoki T; Watanabe K; Kinai E; Honda H; Yazaki H; Tanuma J; Tsukada K; Teruya K; Kikuchi Y; Oka S; Gatanaga H.^124^ | Single-nucleotide polymorphisms in the UDP-glucuronosyltransferase 1A-3' untranslated region are associated with atazanavir-induced nephrolithiasis in patients with HIV-1 infection: a pharmacogenetic study. | 2014 | Other disease group |
| Screen M; Jonson PH; Raheem O; Palmio J; Laaksonen R; Lehtimaki T; Sirito M; Krahe R; Hackman P; Udd B.^125^ | Abnormal splicing of NEDD4 in myotonic dystrophy type 2: possible link to statin adverse reactions. | 2014 | Other disease group |
| Bonora, S., Rusconi, S., Calcagno, A., Bracchi, M., Vigano, O., Cusato, J., . . . Di Perri, G^126^ | Successful pharmacogenetics-based optimization of unboosted atazanavir plasma exposure in HIV-positive patients: a randomized, controlled, pilot study (the REYAGEN study). | 2015 | Other disease group |
| Luo, J. Q., He, F. Z., Wang, Z. M., Sun, N. L., Wang, L. Y., Tang, G. F., . . . Zhang, W.^127^ | SLCO1B1 Variants and Angiotensin Converting Enzyme Inhibitor (Enalapril)-Induced Cough: a Pharmacogenetic Study. | 2015 | Other disease group |
| Ruiz, J., Herrero, M. J., Boso, V., Megias, J. E., Hervas, D., Poveda, J. L., . . . Alino, S. F.^128^ | Impact of Single Nucleotide Polymorphisms (SNPs) on Immunosuppressive Therapy in Lung Transplantation | 2015 | Other disease group |
| Picard, N., Bergan, S., Marquet, P., van Gelder, T., Wallemacq, P., Hesselink, D. A., & Haufroid, V.^129^ | Pharmacogenetic Biomarkers Predictive of the Pharmacokinetics and Pharmacodynamics of Immunosuppressive Drugs. | 2016 | Other disease group |
|  | | | |
| Rohrbacher M; Kirchhof A; Skarke C; Geisslinger G; Lotsch J.^130^ | Rapid identification of three functionally relevant polymorphisms in the OATP1B1 transporter gene using Pyrosequencing. | 2006 | Other dependent variable |
| Hu M; Tomlinson B.^131^ | Effects of statin treatments and polymorphisms in UGT1A1 and SLCO1B1 on serum bilirubin levels in Chinese patients with hypercholesterolaemia. | 2012 | Other dependent variable |
| Tsamandouras, N., Dickinson, G., Guo, Y., Hall, S., Rostami-Hodjegan, A., Galetin, A., & Aarons, L.^132^ | Development and Application of a Mechanistic Pharmacokinetic Model for Simvastatin and its Active Metabolite Simvastatin Acid Using an Integrated Population PBPK Approach. | 2015 | Other dependent variable |

**References**

1. Hedman M, Antikainen M, Holmberg C, Neuvonen M, Eichelbaum M, Kivisto KT*, et al*. Pharmacokinetics and response to pravastatin in paediatric patients with familial hypercholesterolaemia and in paediatric cardiac transplant recipients in relation to polymorphisms of the SLCO1B1 and ABCB1 genes. *Br J Clin Pharmacol* 2006; **61**(6)**:** 706-715.

2. Hermann M, Bogsrud MP, Molden E, Asberg A, Mohebi BU, Ose L*, et al*. Exposure of atorvastatin is unchanged but lactone and acid metabolites are increased several-fold in patients with atorvastatin-induced myopathy. *Clin Pharmacol Ther* 2006; **79**(6)**:** 532-539.

3. Igel M, Arnold KA, Niemi M, Hofmann U, Schwab M, Lutjohann D*, et al*. Impact of the SLCO1B1 polymorphism on the pharmacokinetics and lipid-lowering efficacy of multiple-dose pravastatin. *Clin Pharmacol Ther* 2006; **79**(5)**:** 419-426.

4. Pasanen MK, Neuvonen M, Neuvonen PJ, Niemi M. SLCO1B1 polymorphism markedly affects the pharmacokinetics of simvastatin acid. *Pharmacogenet Genomics* 2006; **16**(12)**:** 873-879.

5. Zhang W, Chen BL, Ozdemir V, He YJ, Zhou G, Peng DD*, et al*. SLCO1B1 521T-->C functional genetic polymorphism and lipid-lowering efficacy of multiple-dose pravastatin in Chinese coronary heart disease patients. *Br J Clin Pharmacol* 2007; **64**(3)**:** 346-352.

6. Couvert P, Giral P, Dejager S, Gu J, Huby T, Chapman MJ*, et al*. Association between a frequent allele of the gene encoding OATP1B1 and enhanced LDL-lowering response to fluvastatin therapy. *Pharmacogenomics* 2008; **9**(9)**:** 1217-1227.

7. Group SC, Link E, Parish S, Armitage J, Bowman L, Heath S*, et al*. SLCO1B1 variants and statin-induced myopathy--a genomewide study. *N Engl J Med* 2008; **359**(8)**:** 789-799.

8. Furihata T, Satoh N, Ohishi T, Ugajin M, Kameyama Y, Morimoto K*, et al*. Functional analysis of a mutation in the SLCO1B1 gene (c.1628T>G) identified in a Japanese patient with pravastatin-induced myopathy. *Pharmacogenomics J* 2009; **9**(3)**:** 185-193.

9. Voora D, Shah SH, Spasojevic I, Ali S, Reed CR, Salisbury BA*, et al*. The SLCO1B1*5 genetic variant is associated with statin-induced side effects. *J Am Coll Cardiol* 2009; **54**(17)**:** 1609-1616.

10. Peters BJ, Rodin AS, Klungel OH, van Duijn CM, Stricker BH, van't Slot R*, et al*. Pharmacogenetic interactions between ABCB1 and SLCO1B1 tagging SNPs and the effectiveness of statins in the prevention of myocardial infarction. *Pharmacogenomics* 2010; **11**(8)**:** 1065-1076.

11. Toms TE, Smith JP, Panoulas VF, Douglas KM, Saratzis AN, Kitas GD. Prevalence of risk factors for statin-induced myopathy in rheumatoid arthritis patients. *Musculoskelet* 2010; **care. 8**(1)**:** 2-9.

12. Donnelly LA, Doney AS, Tavendale R, Lang CC, Pearson ER, Colhoun HM*, et al*. Common nonsynonymous substitutions in SLCO1B1 predispose to statin intolerance in routinely treated individuals with type 2 diabetes: a go-DARTS study. *Clin Pharmacol Ther* 2011; **89**(2)**:** 210-216.

13. Marciante KD, Durda JP, Heckbert SR, Lumley T, Rice K, McKnight B*, et al*. Cerivastatin, genetic variants, and the risk of rhabdomyolysis. *Pharmacogenet Genomics* 2011; **21**(5)**:** 280-288.

14. Rodrigues AC, Perin PM, Purim SG, Silbiger VN, Genvigir FD, Willrich MA*, et al*. Pharmacogenetics of OATP transporters reveals that SLCO1B1 c.388A>G variant is determinant of increased atorvastatin response. *Intj mol sci* 2011; **12**(9)**:** 5815-5827.

15. Santos PC, Soares RA, Nascimento RM, Machado-Coelho GL, Mill JG, Krieger JE*, et al*. SLCO1B1 rs4149056 polymorphism associated with statin-induced myopathy is differently distributed according to ethnicity in the Brazilian general population: Amerindians as a high risk ethnic group. *BMC Med Genet* 2011; **12:** 136.

16. Kaddurah-Daouk R, Baillie RA, Zhu H, Zeng ZB, Wiest MM, Nguyen UT*, et al*. Enteric microbiome metabolites correlate with response to simvastatin treatment.[Erratum appears in PLoS One. 2013;8(5). doi:10.1371/annotation/8e8e95ca-1ac3-4acf-abcb-223cd11ac1c1]. *PLoS ONE* 2011; **6**(10)**:** e25482.

17. Trompet S, de Craen AJ, Postmus I, Ford I, Sattar N, Caslake M*, et al*. Replication of LDL GWAs hits in PROSPER/PHASE as validation for future (pharmaco)genetic analyses. *BMC Med Genet* 2011; **12:** 131.

18. Yang WH, Zeng ZS, Ren XW, Li YP, Shang WJ, Feng GW*, et al*. Simvastatin-induced myopathy with concomitant use of cyclosporine: case report. *International journal of clinical pharmacology and therapeutics* 2011; **49**(12)**:** 772-777.

19. Akao H, Polisecki E, Kajinami K, Trompet S, Robertson M, Ford I*, et al*. Genetic variation at the SLCO1B1 gene locus and low density lipoprotein cholesterol lowering response to pravastatin in the elderly. *Atherosclerosis* 2012; **220**(2)**:** 413-417.

20. Brunham LR, Lansberg PJ, Zhang L, Miao F, Carter C, Hovingh GK*, et al*. Differential effect of the rs4149056 variant in SLCO1B1 on myopathy associated with simvastatin and atorvastatin. *Pharmacogenomics J* 2012; **12**(3)**:** 233-237.

21. Francesca Notarangelo M, Marziliano N, Antonietta Demola M, Pigazzani F, Guidorossi A, Angelica Merlini P*, et al*. Genetic predisposition to atorvastatin-induced myopathy: a case report. *J Clin Pharm Ther* 2012; **37**(5)**:** 604-606.

22. Hubacek JA, Dlouha D, Adamkova V, Lanska V, Ceska R, Vrablik M. Possible gene-gender interaction between the SLCO1B1 polymorphism and statin treatment efficacy. *Neuroendocrinol Lett* 2012; **33 Suppl 2:** 22-25.

23. Santos PC, Gagliardi AC, Miname MH, Chacra AP, Santos RD, Krieger JE*, et al*. SLCO1B1 haplotypes are not associated with atorvastatin-induced myalgia in Brazilian patients with familial hypercholesterolemia. *Eur J Clin Pharmacol* 2012; **68**(3)**:** 273-279.

24. Sortica VA, Fiegenbaum M, Lima LO, Van der Sand CR, Van der Sand LC, Ferreira ME*, et al*. SLCO1B1 gene variability influences lipid-lowering efficacy on simvastatin therapy in Southern Brazilians. *Clin Chem Lab Med* 2012; **50**(3)**:** 441-448.

25. Vrablik M, Hubacek JA, Dlouha D, Lanska V, Rynekrova J, Zlatohlavek L*, et al*. Impact of variants within seven candidate genes on statin treatment efficacy. *Physiol Res* 2012; **61**(6)**:** 609-617.

26. Carr DF, O'Meara H, Jorgensen AL, Campbell J, Hobbs M, McCann G*, et al*. SLCO1B1 genetic variant associated with statin-induced myopathy: a proof-of-concept study using the clinical practice research datalink. *Clin Pharmacol Ther* 2013; **94**(6)**:** 695-701.

27. Danik JS, Chasman DI, MacFadyen JG, Nyberg F, Barratt BJ, Ridker PM. Lack of association between SLCO1B1 polymorphisms and clinical myalgia following rosuvastatin therapy. *Am Heart J* 2013; **165**(6)**:** 1008-1014.

28. Fu Q, Li YP, Gao Y, Yang SH, Lu PQ, Jia M*, et al*. Lack of association between SLCO1B1 polymorphism and the lipid-lowering effects of atorvastatin and simvastatin in Chinese individuals. *Eur J Clin Pharmacol* 2013; **69**(6)**:** 1269-1274.

29. Hopewell JC, Parish S, Offer A, Link E, Clarke R, Lathrop M*, et al*. Impact of common genetic variation on response to simvastatin therapy among 18 705 participants in the Heart Protection Study. *Eur Heart J* 2013; **34**(13)**:** 982-992.

30. Shabana MF, Mishriki AA, Issac MS, Bakhoum SW. Do MDR1 and SLCO1B1 polymorphisms influence the therapeutic response to atorvastatin? A study on a cohort of Egyptian patients with hypercholesterolemia. *Mol Diagn Ther* 2013; **17**(5)**:** 299-309.

31. Tamraz B, Fukushima H, Wolfe AR, Kaspera R, Totah RA, Floyd JS*, et al*. OATP1B1-related drug-drug and drug-gene interactions as potential risk factors for cerivastatin-induced rhabdomyolysis. *Pharmacogenet Genomics* 2013; **23**(7)**:** 355-364.

32. de Keyser CE, Peters BJ, Becker ML, Visser LE, Uitterlinden AG, Klungel OH*, et al*. The SLCO1B1 c.521T>C polymorphism is associated with dose decrease or switching during statin therapy in the Rotterdam Study. *Pharmacogenet Genomics* 2014; **24**(1)**:** 43-51.

33. Drobny M, Pullmann R, Odalos I, Skerenova M, Saniova B. Incidence of skeletal muscle disorders after statins' treatment: consequences in clinical and EMG picture. *Neuroendocrinol Lett* 2014; **35**(2)**:** 123-128.

34. Ferrari M, Guasti L, Maresca A, Mirabile M, Contini S, Grandi AM*, et al*. Association between statin-induced creatine kinase elevation and genetic polymorphisms in SLCO1B1, ABCB1 and ABCG2. *Eur J Clin Pharmacol* 2014; **70**(5)**:** 539-547.

35. Li JH, Joy SV, Haga SB, Orlando LA, Kraus WE, Ginsburg GS*, et al*. Genetically guided statin therapy on statin perceptions, adherence, and cholesterol lowering: a pilot implementation study in primary care patients. *J pers med* 2014; **4**(2)**:** 147-162.

36. Tsamandouras N, Dickinson G, Guo Y, Hall S, Rostami-Hodjegan A, Galetin A*, et al*. Identification of the effect of multiple polymorphisms on the pharmacokinetics of simvastatin and simvastatin acid using a population-modeling approach. *Clin Pharmacol Ther* 2014; **96**(1)**:** 90-100.

37. Birmingham BK, Bujac SR, Elsby R, Azumaya CT, Wei C, Chen Y*, et al*. Impact of ABCG2 and SLCO1B1 polymorphisms on pharmacokinetics of rosuvastatin, atorvastatin and simvastatin acid in Caucasian and Asian subjects: a class effect? *Eur J Clin Pharmacol* 2015; **71**(3)**:** 341-355.

38. Birmingham BK, Bujac SR, Elsby R, Azumaya CT, Zalikowski J, Chen Y*, et al*. Rosuvastatin pharmacokinetics and pharmacogenetics in Caucasian and Asian subjects residing in the United States. *Eur J Clin Pharmacol* 2015; **71**(3)**:** 329-340.

39. Shim H, Chasman DI, Smith JD, Mora S, Ridker PM, Nickerson DA*, et al*. A multivariate genome-wide association analysis of 10 LDL subfractions, and their response to statin treatment, in 1868 Caucasians. *PLoS ONE* 2015; **10**(4)**:** e0120758.

40. Hubacek JA, Dlouha D, Adamkova V, Zlatohlavek L, Viklicky O, Hruba P*, et al*. SLCO1B1 polymorphism is not associated with risk of statin-induced myalgia/myopathy in a Czech population. *Med Sci Monit* 2015; **21:** 1454-1459.

41. Meyer zu Schwabedissen HE, Albers M, Baumeister SE, Rimmbach C, Nauck M, Wallaschofski H*, et al*. Function-impairing polymorphisms of the hepatic uptake transporter SLCO1B1 modify the therapeutic efficacy of statins in a population-based cohort. *Pharmacogenet Genomics* 2015; **25**(1)**:** 8-18.

42. Nagy A, Sipeky C, Szalai R, Melegh BI, Matyas P, Ganczer A*, et al*. Marked differences in frequencies of statin therapy relevant SLCO1B1 variants and haplotypes between Roma and Hungarian populations. *BMC Genet* 2015; **16:** 108.

43. Prado Y, Saavedra N, Zambrano T, Lagos J, Rosales A, Salazar LA. SLCO1B1 c.388A>G Polymorphism Is Associated with HDL-C Levels in Response to Atorvastatin in Chilean Individuals. *Int j mol sci* 2015; **16**(9)**:** 20609-20619.

44. Kadam P, Ashavaid TF, Ponde CK, Rajani RM. Genetic determinants of lipid-lowering response to atorvastatin therapy in an Indian population. *J Clin Pharm Ther* 2016; **41**(3)**:** 329-333.

45. Sychev DA, Shuev GN, Chertovskih JV, Maksimova NR, Grachev AV, Syrkova OA. The frequency of SLCO1B1*5 polymorphism genotypes among Russian and Sakha (Yakutia) patients with hypercholesterolemia. *Pharmgenomics pers med* 2016; **9:** 59-63.

46. Romaine SP, Bailey KM, Hall AS, Balmforth AJ. The influence of SLCO1B1 (OATP1B1) gene polymorphisms on response to statin therapy. *Pharmacogenomics J* 2010; **10**(1)**:** 1-11.

47. Generaux GT, Bonomo FM, Johnson M, Doan KM. Impact of SLCO1B1 (OATP1B1) and ABCG2 (BCRP) genetic polymorphisms and inhibition on LDL-C lowering and myopathy of statins. *Xenobiotica* 2011; **41**(8)**:** 639-651.

48. Maggo SD, Kennedy MA, Clark DW. Clinical implications of pharmacogenetic variation on the effects of statins. *Drug Saf* 2011; **34**(1)**:** 1-19.

49. Wang P. Statin dose in Asians: is pharmacogenetics relevant? *Pharmacogenomics* 2011; **12**(11)**:** 1605-1615.

50. Cardelli M, Marchegiani F, Corsonello A, Lattanzio F, Provinciali M. A review of pharmacogenetics of adverse drug reactions in elderly people. *Drug Saf* 2012; **35 Suppl 1:** 3-20.

51. Daly AK. Using genome-wide association studies to identify genes important in serious adverse drug reactions. *Annu Rev Pharmacol Toxicol* 2012; **52:** 21-35.

52. Feng Q, Wilke RA, Baye TM. Individualized risk for statin-induced myopathy: current knowledge, emerging challenges and potential solutions. *Pharmacogenomics* 2012; **13**(5)**:** 579-594.

53. Ong FS, Deignan JL, Kuo JZ, Bernstein KE, Rotter JI, Grody WW*, et al*. Clinical utility of pharmacogenetic biomarkers in cardiovascular therapeutics: a challenge for clinical implementation. *Pharmacogenomics* 2012; **13**(4)**:** 465-475.

54. Scarpini F, Cappellone R, Auteri A, Puccetti L. Role of genetic factors in statins side-effects. *Cardiovasc Hematol Disord Drug Targets* 2012; **12**(1)**:** 35-43.

55. Sirtori CR, Mombelli G, Triolo M, Laaksonen R. Clinical response to statins: mechanism(s) of variable activity and adverse effects. *Ann Med* 2012; **44**(5)**:** 419-432.

56. Voora D, Ginsburg GS. Clinical application of cardiovascular pharmacogenetics. *J Am Coll Cardiol* 2012; **60**(1)**:** 9-20.

57. Wells QS, Delaney JT, Roden DM. Genetic determinants of response to cardiovascular drugs. *Curr Opin Cardiol* 2012; **27**(3)**:** 253-261.

58. Wilke RA, Ramsey LB, Johnson SG, Maxwell WD, McLeod HL, Voora D*, et al*. The clinical pharmacogenomics implementation consortium: CPIC guideline for SLCO1B1 and simvastatin-induced myopathy. *Clin Pharmacol Ther* 2012; **92**(1)**:** 112-117.

59. Johnson JA, Cavallari LH. Pharmacogenetics and cardiovascular disease--implications for personalized medicine. *Pharmacol Rev* 2013; **65**(3)**:** 987-1009.

60. Kitzmiller JP, Binkley PF, Pandey SR, Suhy AM, Baldassarre D, Hartmann K. Statin pharmacogenomics: pursuing biomarkers for predicting clinical outcomes. *Discov medicin* 2013; **16**(86)**:** 45-51.

61. Sorich MJ, Wiese MD, O'Shea RL, Pekarsky B. Review of the cost effectiveness of pharmacogenetic-guided treatment of hypercholesterolaemia. *Pharmacoeconomics* 2013; **31**(5)**:** 377-391.

62. Stewart A. SLCO1B1 Polymorphisms and Statin-Induced Myopathy. *PLOS Current Evidence on Genomic Tests* 2013;(December).

63. Yip VL, Pirmohamed M. Expanding role of pharmacogenomics in the management of cardiovascular disorders. *Am J Cardiovasc Drugs* 2013; **13**(3)**:** 151-162.

64. Desai CS, Martin SS, Blumenthal RS. Non-cardiovascular effects associated with statins. *BMJ* 2014; **349:** g3743.

65. Gelissen IC, McLachlan AJ. The pharmacogenomics of statins. *Pharmacol Res* 2014; **88:** 99-106.

66. Hopewell JC, Reith C, Armitage J. Pharmacogenomics of statin therapy: any new insights in efficacy or safety? *Curr Opin Lipidol* 2014; **25**(6)**:** 438-445.

67. Needham M, Mastaglia FL. Statin myotoxicity: a review of genetic susceptibility factors. *Neuromuscul Disord* 2014; **24**(1)**:** 4-15.

68. Norata GD, Tibolla G, Catapano AL. Statins and skeletal muscles toxicity: from clinical trials to everyday practice. *Pharmacol Res* 2014; **88:** 107-113.

69. Postmus I, Trompet S, Deshmukh HA, Barnes MR, Li X, Warren HR*, et al*. Pharmacogenetic meta-analysis of genome-wide association studies of LDL cholesterol response to statins. *Nat Commun* 2014; **5:** 5068.

70. Ramsey LB, Johnson SG, Caudle KE, Haidar CE, Voora D, Wilke RA*, et al*. The clinical pharmacogenetics implementation consortium guideline for SLCO1B1 and simvastatin-induced myopathy: 2014 update. *Clin Pharmacol Ther* 2014; **96**(4)**:** 423-428.

71. Sissung TM, Goey AK, Ley AM, Strope JD, Figg WD. Pharmacogenetics of membrane transporters: a review of current approaches. *Methods Mol Biol* 2014; **1175:** 91-120.

72. Tompkins R, Schwartzbard A, Gianos E, Fisher E, Weintraub H. A current approach to statin intolerance. *Clin Pharmacol Ther* 2014; **96**(1)**:** 74-80.

73. Turner RM, Pirmohamed M. Cardiovascular pharmacogenomics: expectations and practical benefits. *Clin Pharmacol Ther* 2014; **95**(3)**:** 281-293.

74. Weeke P, Roden DM. Applied pharmacogenomics in cardiovascular medicine. *Annu Rev Med* 2014; **65:** 81-94.

75. Dai R, Feng J, Wang Y, Yang Y, Deng C, Tang X*, et al*. Association between SLCO1B1 521 T>C and 388 A>G Polymorphisms and Statins Effectiveness: A Meta-Analysis. *J Atheroscler Thromb* 2015; **22**(8)**:** 796-815.

76. Dou Y, Zhu X, Wang Q, Tian X, Cheng J, Zhang E. Meta-Analysis of the SLCO1B1 c.521T>C Variant Reveals Slight Influence on the Lipid-Lowering Efficacy of Statins. *Ann Lab Med* 2015; **35**(3)**:** 329-335.

77. Hou Q, Li S, Li L, Li Y, Sun X, Tian H. Association Between SLCO1B1 Gene T521C Polymorphism and Statin-Related Myopathy Risk: A Meta-Analysis of Case-Control Studies. *Medicine* 2015; **94**(37)**:** e1268.

78. Patel J, Superko HR, Martin SS, Blumenthal RS, Christopher-Stine L. Genetic and immunologic susceptibility to statin-related myopathy. *Atherosclerosis* 2015; **240**(1)**:** 260-271.

79. Kitzmiller JP, Mikulik EB, Dauki AM, Murkherjee C, Luzum JA. Pharmacogenomics of statins: understanding susceptibility to adverse effects. *Pharmgenomics pers med* 2016; **9:** 97-106.

80. Leusink M, Onland-Moret NC, de Bakker PI, de Boer A, Maitland-van der Zee AH. Seventeen years of statin pharmacogenetics: a systematic review. *Pharmacogenomics* 2016; **17**(2)**:** 163-180.

81. Tonk EC, Gurwitz D, Maitland-van der Zee AH, Janssens AC. Assessment of pharmacogenetic tests: presenting measures of Sensitivity, Specificity, PPV, NPV and potential population impact in association studies. *Pharmacogenomics J* 2016;(May 10.).

82. Nakamura Y. Pharmacogenomics and drug toxicity. *N Engl J Med* 2008; **359**(8)**:** 856-858.

83. Mitka M. Researchers worry about myopathy risk for patients taking high-dose simvastatin. *JAMA* 2009; **301**(3)**:** 261-262.

84. Rossi JS, McLeod HL. The pharmacogenetics of statin therapy: when the body aches, the mind will follow. *J Am Coll Cardiol* 2009; **54**(17)**:** 1617-1618.

85. Vladutiu GD, Isackson PJ. SLCO1B1 variants and statin-induced myopathy. *N Engl J Med* 2009; **360**(3)**:** 304.

86. Puccetti L, Ciani F, Auteri A. Genetic involvement in statins induced myopathy. Preliminary data from an observational case-control study. *Atherosclerosis* 2010; **211**(1)**:** 28-29.

87. Becquemont L, Alfirevic A, Amstutz U, Brauch H, Jacqz-Aigrain E, Laurent-Puig P*, et al*. Practical recommendations for pharmacogenomics-based prescription: 2010 ESF-UB Conference on Pharmacogenetics and Pharmacogenomics. *Pharmacogenomics* 2011; **12**(1)**:** 113-124.

88. Couvert P, Chapman MJ, Carrie A. Impact of genetic variation in the SLCO1B1 gene on statin efficacy in low-density lipoprotein cholesterol-lowering therapy. *Pharmacogenomics* 2011; **12**(2)**:** 137-139.

89. Dolgin E. Pharmacogenetic tests yield bonus benefit: better drug adherence. *Nat Med* 2013; **19**(11)**:** 1354-1355.

90. Rosskopf D, Meyer zu Schwabedissen HE, Kroemer HK, Siegmund W. [Pharmacogenomics in routine medical care]. *Dtsch Med Wochenschr* 2010; **135**(4)**:** 133-144; quiz 145-136.

91. Dendramis G. [Interindividual differences in the response to statin therapy and gene polymorphisms related to myopathy during statin therapy]. *G ital cardiol* 2011; **12**(3)**:** 182-185.

92. Saito Y, Sawada J. [Pharmacogenomics]. *Nippon Rinsho* 2009; **67**(6)**:** 1175-1179.

93. Chiba K, Morimoto K. [Genetic marker of statin-induced rhabdomyolysis]. *Yakugaku Zasshi* 2011; **131**(2)**:** 247-253.

94. Garcia-Sabina A, Gulin-Davila J, Sempere-Serrano P, Gonzalez-Juanatey C, Martinez-Pacheco R. [Specific considerations on the prescription and therapeutic interchange of statins]. *FARM HOSP* 2012; **36**(2)**:** 97-108.

95. Garcia-Estevez DA, San Millan B, Navarro C, Sogo T. [Myopathy due to deficiency of desaminase myoadenilate induced by atorvastatine]. *Med Clin* 2013; **140**(12)**:** 565-567.

96. Garwicz D, Wadelius M. [Pharmacogenetic analysis can predict adverse effects of statins]. *Lakartidningen* 2013; **110**(19-20)**:** 951-952.

97. Anonymous. Drug interactions with simvastatin. *Med Lett Drugs Ther* 2008; **50**(1297)**:** 83-84.

98. Deng JW, Shon JH, Shin HJ, Park SJ, Yeo CW, Zhou HH*, et al*. Effect of silymarin supplement on the pharmacokinetics of rosuvastatin. *Pharm Res* 2008; **25**(8)**:** 1807-1814.

99. Shin KH, Kim TE, Kim SE, Lee MG, Song IS, Yoon SH*, et al*. The effect of the newly developed angiotensin receptor II antagonist fimasartan on the pharmacokinetics of atorvastatin in relation to OATP1B1 in healthy male volunteers. *J Cardiovasc Pharmacol* 2011; **58**(5)**:** 492-499.

100. Elsby R, Hilgendorf C, Fenner K. Understanding the critical disposition pathways of statins to assess drug-drug interaction risk during drug development: it's not just about OATP1B1. *Clin Pharmacol Ther* 2012; **92**(5)**:** 584-598.

101. Hua WJ, Hua WX, Fang HJ. The role of OATP1B1 and BCRP in pharmacokinetics and DDI of novel statins. *Cardiovasc Ther* 2012; **30**(5)**:** e234-241.

102. Marusic S, Lisicic A, Horvatic I, Bacic-Vrca V, Bozina N. Atorvastatin-related rhabdomyolysis and acute renal failure in a genetically predisposed patient with potential drug-drug interaction. *Int J Clin Pharm* 2012; **34**(6)**:** 825-827.

103. Custodio JM, Wang H, Hao J, Lepist EI, Ray AS, Andrews J*, et al*. Pharmacokinetics of cobicistat boosted-elvitegravir administered in combination with rosuvastatin. *J Clin Pharmacol* 2014; **54**(6)**:** 649-656.

104. Kellick KA, Bottorff M, Toth PP, The National Lipid Association's Safety Task F. A clinician's guide to statin drug-drug interactions. *J clin lipidol* 2014; **8**(3 Suppl)**:** S30-46.

105. Kulmatycki K, Hanna I, Meyers D, Salunke A, Movva A, Majumdar T*, et al*. Evaluation of a potential transporter-mediated drug interaction between rosuvastatin and pradigastat, a novel DGAT-1 inhibitor. *International journal of clinical pharmacology and therapeutics* 2015; **53**(5)**:** 345-355.

106. Li DQ, Kim R, McArthur E, Fleet JL, Bailey DG, Juurlink D*, et al*. Risk of adverse events among older adults following co-prescription of clarithromycin and statins not metabolized by cytochrome P450 3A4. *CMAJ : Canadian Medical Association journal = journal de l'Association medicale canadienne* 2015; **187**(3)**:** 174-180.

107. Howe K, Sanat F, Thumser AE, Coleman T, Plant N. The statin class of HMG-CoA reductase inhibitors demonstrate differential activation of the nuclear receptors PXR, CAR and FXR, as well as their downstream target genes. *Xenobiotica* 2011; **41**(7)**:** 519-529.

108. Bjorkhem-Bergman L, Bergstrom H, Johansson M, Parini P, Eriksson M, Rane A*, et al*. Atorvastatin treatment induces uptake and efflux transporters in human liver. *Drug Metab Dispos* 2013; **41**(9)**:** 1610-1615.

109. Hu M, Mak VW, Yin OQ, Chu TT, Tomlinson B. Effects of grapefruit juice and SLCO1B1 388A>G polymorphism on the pharmacokinetics of pitavastatin. *Drug metab* 2013; **pharmacokineti.. 28**(2)**:** 104-108.

110. Stojakovic N, Igic R. Simvastatin-lnduced nocturnal leg pain disappears with pravastatin substitution. *Srp Arh Celok Lek* 2013; **141**(5-6)**:** 387-389.

111. Apostolopoulou M, Corsini A, Roden M. The role of mitochondria in statin-induced myopathy. *Eur J Clin Invest* 2015; **45**(7)**:** 745-754.

112. Werner D, Werner U, Meybaum A, Schmidt B, Umbreen S, Grosch A*, et al*. Determinants of steady-state torasemide pharmacokinetics: impact of pharmacogenetic factors, gender and angiotensin II receptor blockers. *Clin Pharmacokinet* 2008; **47**(5)**:** 323-332.

113. Han JY, Lim HS, Park YH, Lee SY, Lee JS. Integrated pharmacogenetic prediction of irinotecan pharmacokinetics and toxicity in patients with advanced non-small cell lung cancer. *Lung Cancer* 2009; **63**(1)**:** 115-120.

114. Innocenti F, Kroetz DL, Schuetz E, Dolan ME, Ramirez J, Relling M*, et al*. Comprehensive pharmacogenetic analysis of irinotecan neutropenia and pharmacokinetics. *J Clin Oncol* 2009; **27**(16)**:** 2604-2614.

115. Ni W, Ji J, Dai Z, Papp A, Johnson AJ, Ahn S*, et al*. Flavopiridol pharmacogenetics: clinical and functional evidence for the role of SLCO1B1/OATP1B1 in flavopiridol disposition. *PLoS ONE* 2010; **5**(11)**:** e13792.

116. Di Paolo A, Bocci G, Polillo M, Del Re M, Di Desidero T, Lastella M*, et al*. Pharmacokinetic and pharmacogenetic predictive markers of irinotecan activity and toxicity. *Curr Drug Metab* 2011; **12**(10)**:** 932-943.

117. Yimer G, Ueda N, Habtewold A, Amogne W, Suda A, Riedel KD*, et al*. Pharmacogenetic & pharmacokinetic biomarker for efavirenz based ARV and rifampicin based anti-TB drug induced liver injury in TB-HIV infected patients. *PLoS ONE* 2011; **6**(12)**:** e27810.

118. Kile DA, MaWhinney S, Aquilante CL, Rower JE, Castillo-Mancilla JR, Anderson PL. A population pharmacokinetic-pharmacogenetic analysis of atazanavir. *AIDS Res Hum Retroviruses* 2012; **28**(10)**:** 1227-1234.

119. Shiotani A, Murao T, Sakakibara T, Tarumi K, Manabe N, Kamada T*, et al*. Association of SLCO1B1 1b with peptic ulcer amongst Japanese patients taking low-dose aspirin. *Dig Liver Dis* 2012; **44**(3)**:** 201-205.

120. De Mattia E, Toffoli G, Polesel J, D'Andrea M, Corona G, Zagonel V*, et al*. Pharmacogenetics of ABC and SLC transporters in metastatic colorectal cancer patients receiving first-line FOLFIRI treatment. *Pharmacogenet Genomics* 2013; **23**(10)**:** 549-557.

121. Huang L, Zhang T, Xie C, Liao X, Yu Q, Feng J*, et al*. SLCO1B1 and SLC19A1 gene variants and irinotecan-induced rapid response and survival: a prospective multicenter pharmacogenetics study of metastatic colorectal cancer. *PLoS ONE* 2013; **8**(10)**:** e77223.

122. Radtke S, Zolk O, Renner B, Paulides M, Zimmermann M, Moricke A*, et al*. Germline genetic variations in methotrexate candidate genes are associated with pharmacokinetics, toxicity, and outcome in childhood acute lymphoblastic leukemia. *Blood* 2013; **121**(26)**:** 5145-5153.

123. Han N, Yun HY, Kim IW, Oh YJ, Kim YS, Oh JM. Population pharmacogenetic pharmacokinetic modeling for flip-flop phenomenon of enteric-coated mycophenolate sodium in kidney transplant recipients. *Eur J Clin Pharmacol* 2014; **70**(10)**:** 1211-1219.

124. Nishijima T, Tsuchiya K, Tanaka N, Joya A, Hamada Y, Mizushima D*, et al*. Single-nucleotide polymorphisms in the UDP-glucuronosyltransferase 1A-3' untranslated region are associated with atazanavir-induced nephrolithiasis in patients with HIV-1 infection: a pharmacogenetic study. *J Antimicrob Chemother* 2014; **69**(12)**:** 3320-3328.

125. Screen M, Jonson PH, Raheem O, Palmio J, Laaksonen R, Lehtimaki T*, et al*. Abnormal splicing of NEDD4 in myotonic dystrophy type 2: possible link to statin adverse reactions. *Am J Pathol* 2014; **184**(8)**:** 2322-2332.

126. Bonora S, Rusconi S, Calcagno A, Bracchi M, Vigano O, Cusato J*, et al*. Successful pharmacogenetics-based optimization of unboosted atazanavir plasma exposure in HIV-positive patients: a randomized, controlled, pilot study (the REYAGEN study). *J Antimicrob Chemother* 2015; **70**(11)**:** 3096-3099.

127. Luo JQ, He FZ, Wang ZM, Sun NL, Wang LY, Tang GF*, et al*. SLCO1B1 Variants and Angiotensin Converting Enzyme Inhibitor (Enalapril)-Induced Cough: a Pharmacogenetic Study. *Scientific reports* 2015; **5:** 17253.

128. Ruiz J, Herrero M, Bosó V, Megías JE, Hervás D, Poveda JL*, et al*. Impact of Single Nucleotide Polymorphisms (SNPs) on Immunosuppressive Therapy in Lung Transplantation. *International Journal of Molecular Sciences* 2015; **16**(9)**:** 20168-20182.

129. Picard N, Bergan S, Marquet P, van Gelder T, Wallemacq P, Hesselink DA*, et al*. Pharmacogenetic Biomarkers Predictive of the Pharmacokinetics and Pharmacodynamics of Immunosuppressive Drugs. *Therapeutic drug monitoring* 2016; **38 Suppl 1:** S57-69.

130. Rohrbacher M, Kirchhof A, Skarke C, Geisslinger G, Lotsch J. Rapid identification of three functionally relevant polymorphisms in the OATP1B1 transporter gene using Pyrosequencing. *Pharmacogenomics* 2006; **7**(2)**:** 167-176.

131. Hu M, Tomlinson B. Effects of statin treatments and polymorphisms in UGT1A1 and SLCO1B1 on serum bilirubin levels in Chinese patients with hypercholesterolaemia. *Atherosclerosis* 2012; **223**(2)**:** 427-432.

132. Tsamandouras N, Dickinson G, Guo Y, Hall S, Rostami-Hodjegan A, Galetin A*, et al*. Development and Application of a Mechanistic Pharmacokinetic Model for Simvastatin and its Active Metabolite Simvastatin Acid Using an Integrated Population PBPK Approach. *Pharm Res* 2015; **32**(6)**:** 1864-1883.
